# An improved hyperparameter optimization framework for AutoML systems using evolutionary algorithms

**DOI:** 10.1038/s41598-023-32027-3

**Published:** 2023-03-23

**Authors:** Amala Mary Vincent, P. Jidesh

**Affiliations:** Department of Mathematical and Computational Sciences, National Institute of Technology Karnataka, Mangalore, 575025 India

**Keywords:** Engineering, Mathematics and computing

## Abstract

For any machine learning model, finding the optimal hyperparameter setting has a direct and significant impact on the model’s performance. In this paper, we discuss different types of hyperparameter optimization techniques. We compare the performance of some of the hyperparameter optimization techniques on image classification datasets with the help of AutoML models. In particular, the paper studies Bayesian optimization in depth and proposes the use of genetic algorithm, differential evolution and covariance matrix adaptation—evolutionary strategy for acquisition function optimization. Moreover, we compare these variants of Bayesian optimization with conventional Bayesian optimization and observe that the use of covariance matrix adaptation—evolutionary strategy and differential evolution improves the performance of standard Bayesian optimization. We also notice that Bayesian optimization tends to perform poorly when genetic algorithm is used for acquisition function optimization.

## Introduction

Machine learning strategy has changed the face of automated models by integrating themselves into many application domains. The spectrum of applications ranges over various domains, from atmospheric analysis to medical diagnosis. All these applications are design-sensitive, implying that the model’s performance depends highly on the selected machine learning algorithm, training procedures, regularization methods, and most importantly, on selecting the optimal set of hyperparameters.

Every machine learning model has two types of parameters, a set that is trained by the model and another that controls the learning process. The former set of parameters is determined using the training dataset and is called the model parameters. The latter are values that can be tuned and adjusted by the user before running the model. They have a major role in determining the performance of the model and are called hyperparameters. The weights of a neural network are model parameters that are derived and fitted by training, whereas the learning rate of a neural network, the regularization parameter, and the kernel parameter are all examples of hyperparameters. Different machine learning algorithms require various sets of hyperparameters. Other than a few simple models like least square regression, most machine learning models have hyperparameters.

Different hyperparameter configurations are required for different datasets. It is important to find a hyperparameter setting that performs optimally for a given algorithm trained over a particular dataset. This is done by tuning the hyperparameters and the technique is called Hyperparameter Optimization (HPO)^1^. Once we have the hyperparameters, the algorithm learns the model parameters from the data.

Every machine learning algorithm aims to identify a function that optimizes the loss. Let *M* be the given machine learning algorithm with $\{\lambda _1, \lambda _2,\ldots \lambda _n\}$ the parameters to be tuned. Each hyperparameter $\lambda _i$ can have a value within the interval $[a_i, b_i]$ in a hyperparameter configuration space $\Lambda = [a_1, b_1] \times \cdots \times [a_n, b_n]$. Here *F* is a function that determines the performance or loss value. Further, *F* maps each possible configuration $\lambda \in \Lambda$ to a numerical value $F:\Lambda \rightarrow R^{*}$. The objective of hyperparameter optimization is to find the best configuration $\lambda ^{*}$ that minimizes $F(\lambda )$.

1 $$\begin{aligned} \lambda ^{*} = \mathop {\textrm{argmin}}\limits _{\lambda \in \Lambda } F(\lambda ) \end{aligned}$$

The objective function *F* is a black-box function, meaning the actual function is unknown, but one can observe its output based on specific given inputs. Since the function cannot be accessed, we do not have any information about its derivative. The function evaluation is pretty time-consuming, implying that each iteration takes a considerable amount of time. This can be minutes for a small-scale dataset, while it can be hours and days for larger ones. As a result, solving the optimization problem is difficult, and obtaining the ideal configuration in a few trials necessitates a specific approach.

Some of the popular HPO methods are grid search and random search, Bayesian optimization (BO), gradient descent, evolution-based techniques and multi-fidelity methods like hyperband and successive halving.

An extensive analysis of existing HPO techniques is presented in the paper. Considering various advantages of Bayesian optimization in solving black-box optimization problems, we look into ways to improve the conventional BO with the help of evolutionary algorithms. We have used evolutionary algorithms, taking into account their success in solving optimization problems and major advantages like ease of implementation, robustness and parallelizability. The paper also delivers an empirical comparison of the application of evolutionary algorithms for acquisition function optimization.

This paper is organized as follows. First, we discuss previous works on HPO and the existing AutoML models in detail. Then, we highlight the motivation for a new model. In the subsequent section, we compare evolutionary algorithms for acquisition function optimization. Finally, we examine the results and conclude the paper.

## A comprehensive analysis of previous works

### Hyperparameter optimization

The first and basic approach put forward for performing HPO was grid search. Grid search performs an exhaustive search through the Cartesian product of manually specified, finite sets of hyperparameters^2,3^. It is time-consuming and endures the problem of dimensionality. Random search proves to be more efficient than grid search in high-dimensional spaces^4^. It selects the combination of hyperparameters from the search space randomly.

Sequential Model-Based Optimization (SMBO) is a formalization of Bayesian Optimization (BO)^5–11^. The BO approach treats the black-box objective function as a random function and assumes a prior distribution over the loss function which is updated from new observations to a posterior distribution. In other words, it constructs a probabilistic model that maps the hyperparameters to a probability score, denoted as *p*(*x*|*y*). This model is a surrogate for the expensive-to-evaluate objective function. SMBO is a sequential model that runs multiple trials one after another, finding a more promising set of hyperparameters each time^12,13^. The criterion used for selecting the next best hyperparameter values is called an acquisition function, which uses the surrogate function information to obtain the next point to be evaluated. These hyperparameter values are then used to evaluate the objective function and the (probability score, hyperparameter) pairs are finally used to update the surrogate model.

Mostly used surrogate models are Gaussian Process (GP), Random Forest Regressions, and Tree Parzen Estimator (TPE) while the most preferred choice for acquisition function is Expected Improvement. Unlike grid search and random search, SMBO keeps track of past evaluation results. Even though the method is inherently serial and difficult to parallelize, it runs faster than random search^14^.

One of the major limitations of SMBO is the uncertainty regarding the choice of acquisition function. Since it is a sequential model, achieving parallelization is almost futile. Another issue is that the expense for different data varies significantly as the function evaluation step is a laborious procedure.

Further, there is another class of algorithms that are population-based, nature-inspired metaheuristic approaches. The term metaheuristics come from two words, meta meaning “beyond” and heuristics meaning “to find”. Metaheuristics are an algorithmic framework that efficiently modifies domain-specific knowledge into heuristics to produce better solutions^15,16^. This modification generally involves two procedures: exploration (diversification) and exploitation (intensification) and is done with the help of heuristic operators. These heuristic operators are usually inspired by nature and are different in different evolutionary algorithms. Diversification aims to cover as much of the search area as feasible, whereas intensification aims to fully exploit the search space in order to arrive at better solutions as rapidly as possible.

Like all other optimization algorithms, this family of algorithms also try to optimize the objective function in an iterative fashion, by updating the parameter (solution) values. This can be represented by the equation, $y_i=y_{i-1}+ \Delta y_{i-1}$ where *i* indicates the number of iterations, then $y_{i-1}$ is the solution vector in the previous iteration, $y_i$ is the new vector of solutions, and $\Delta y_{i-1}$ signifies the change in the solution vector after one iteration. Based on how $\Delta y_{i-1}$ is determined, there are two general categories of algorithms: “Evolutionary Algorithms (EA)” and “Swarm Intelligence (SI)”. The former includes algorithms like Genetic Algorithms (GA), Genetic Programming, Evolutionary Strategies (ES), Evolutionary Programming etc., that are based on biological evolution, with selection, crossover (recombination), and mutation phases. EAs have been proven effective in finding good hyperparameter settings for a wide range of ML problems and are particularly useful when the search space is large and complex or when the objective function is noisy or expensive to evaluate^17^. SI includes Particle Swarm Optimization and Ant Colony Optimization and is influenced by the social conduct of natural organisms like birds, fish and ants^18,19^.

GA are a class of evolutionary algorithms, hence both iterative and population-based^20^. On each iteration, they work with several solutions collectively called a population rather than a single solution. A GA initialises a random population as the solution and updates this solution with the help of three operators, namely, selection, crossover and mutation (variation operators). This is continued until the stopping criteria is met. A single iteration is designated as one generation of GA. The term genetic algorithm comes from the similarity of the representation of solutions to chromosomes and that of GA operators to genetic operators. GA has been used to find optimal hyperparameter settings for many ML problems^21,22^.

Particle Swarm Optimization (PSO) is an algorithm inspired by the behaviour of fish and birds moving in a group^23,24^. Ant Colony Optimization (ACO) is another category of swarm intelligence algorithms which is influenced by the nature of ant colonies searching for food^25,26^. Both of these algorithms have been used for hyperparameter optimization of different ML models^27,28^.

Grid search, random search and population-based methods like the Covariance Matrix Adaptation—Evolutionary Strategy^29^ (CMA-ES) are the common model-free paradigms used for hyperparameter tuning in AutoML systems. Apart from these model-free black-box optimization techniques, Bayesian optimization, HPO in AutoML focuses on multi-fidelity methods that are cheaper compared to the pure black-box methods. This includes an early stopping algorithm, a simple selection algorithm and other adaptive choices of fidelity.

A major hurdle in the optimization procedure is the exhaustive runtime of the algorithms which increases with the amount of dataset and number of hyperparameters. Multi-fidelity methods focus on finding a solution to the time and resource constraints in HPO. Reducing the data set for training and bringing down the number of features, iterations, and cross-validation folds are a few manual approaches used to accelerate the tuning process. These ideas are used by low fidelity methods to find an approximation of the actual objective function to minimise.

Learning curve-based prediction is one such method that gets rid of configurations that are anticipated to perform badly^30^. It’s an early stopping algorithm that examines the learning curve during HPO and stops the training operation for a certain hyperparameter setting if the curve isn’t expected to meet the performance level of the best model produced up until that point in the optimization process. Implementation of these models combined with the Bayesian optimization technique called the Freeze–Thaw Bayesian optimization is mentioned in Swersky et al.^31^. The algorithm maintains a set of frozen configurations and uses an information-theoretic decision framework to either thaw (defreeze) a setting chosen by the Bayesian optimization and continue training or train a new configuration to find the best hyperparameter settings.

Selection algorithms like Successive Halving (SH) and Hyperbands are bandit-based strategies that are pure-exploration focused resource allocation problems^32^. Pure exploration problems are also called best-arm identification problems. The goal is to choose the best arm (here, the best settings with the maximum expected performance) with maximum confidence. Successive halving is one such non-stochastic best-arm identification problem proposed by Jamieson et al.^33^. The algorithm can be summarized in 3 steps. (1) At first, it uniformly allocates resources to each set of hyperparameter configurations. (2) And then assess how they perform. (3) Lastly, the algorithm removes half of the worst-performing group. The process is iterated till a single configuration remains. Through the sequential elimination step, the algorithm guarantees more resources for more reliable configurations^34^.

Hyperband is an extension of the successive halving algorithm put forward by Li et al.^35^. In successive halving, the fixed budget of resources is uniformly distributed to all configurations initially. If there are n configurations and a total budget B of resources, then SH allocates B/n resources for each setting. Whereas hyperband takes into consideration the fact that a large number of the configurations (large n) will require only a small amount of resource (small B/n) or vice versa. This is called the n versus B/n issue. Hyperband addresses this by letting different possible values of n for a fixed B and allocating a minimum resource r for all the configurations before the elimination step. And then calls SH on random samples of configurations as a subroutine^36^.

The multi-task Bayesian optimization technique is an adaptive fidelity technique that learns from previously trained models or a trained subset of a large dataset^37^. They use multi-task Gaussian process models for fastening the Bayesian optimization by studying the correlation among tasks. Other adaptive choices of fidelity include algorithms like Bayesian Optimization with Hyperband (BOHB)^38^, Multi-Fidelity Gaussian Process Upper Confidence Bound (MFGP-UCB)^39^ and Bayesian optimization with Continuous Approximations (BOCA)^40^. BOCA employs an Upper Confidence Bound (UCB) acquisition function to aid the optimization process. Apart from the above mentioned methods, there are many more recent works on HPO on different applications^41–47^. Direct search class of black-box optimization methods have also been adapted to HPO of deep ML models^48,49^.

### AutoML systems

Different ML models are appropriate for different applications. In order to find the most suitable algorithm, the simple method of applying and optimizing all known learning algorithms is not practical in most cases. This process of finding the best ML algorithm and creating the optimal architecture by setting the best hyperparameters is a complex and time-consuming process. Here comes the importance of an Automated Machine Learning (AutoML) system that determines the optimal configurations for a particular application with the best performance within the time constraints. For a deep learning network, AutoML not only performs Hyperparameter Optimization (HPO) to automatically set the optimal hyperparameters but also selects the right neural architecture for each layer. It also provides tools and approaches for enabling ML to be accessible to non-experts, increasing performance, and speeding up ML research.

AutoML systems automate the end-to-end process of machine learning involving automating 4 phases—data preparation, feature processing, model generation and estimation. The user only needs to submit data, and the AutoML system will automatically decide which strategy is optimal for a particular application. The primary goal of AutoML systems is to optimise the performance by automatically setting the best hyperparameters, i.e., automating the hyperparameter optimization part. It also has several other use cases. It decreases the amount of human labour required to apply machine learning, increases the effectiveness of machine learning algorithms, and makes scientific investigations more reproducible. Some of the popular AutoML frameworks and the optimizers they use are listed below:

1. ML models-based frameworks
  - Auto-WEKA Auto-WEKA is a fully automated Automl system that includes feature selection. It uses a Bayesian optimization framework for HPO^50^.
  - Auto-sklearn Auto-sklearn has feature processing and pre-processing units. It considers 15 classifiers and 110 hyperparameters and considers previous performances on similar datasets. Auto-sklearn can also construct ensemble models using a TPE-based BO for HPO^51^.
  - Tree-based Pipeline Optimization Tool (TPOT) It is a genetic programming-based AutoML. TPOT optimizes a sequence of feature preprocessors and machine learning models to enhance the classification accuracy by making use of GA for hyperparameter tuning^52^.
2. DL models-based frameworks
  - AutoKeras AutoKeras focuses on deep learning tasks and uses BO to guide neural architecture search. It also employs a neural network kernel and a tree-structured acquisition function optimization technique to effectively evaluate the search space^53^.
  - Auto-PyTorch Auto-PyTorch is a Neural Architecture Search (NAS) library based on the same principles as AutoKeras, but with a PyTorch backend. It employs BO combined with hyperband for HPO^54^.
  - AutoGluon AutoGluon uses multi-layer ensemble models and performs complex data processing and deep learning^55^.
  - H2O AutoML Make use of one layer of ensemble stacking combined with bagging and a strong base model—XGBoost tree ensemble^56^. H2O uses random search for hyperparameter tuning.
3. Hyperparameter tuners Packages that use Bayesian optimization include SMAC^57,58^, Spearmint, Hyperopt^59^, Scikit-optimize, BoTorch etc. Packages like DEHB^60^, DEAP^61^ and Nevergrad make use of evolutionary algorithms, whereas Optuna^62^, Orion and RayTune implement both Bayesian optimization and evolutionary algorithms.

## Motivation for a new model

Hyperparameter optimization is an important and ubiquitous problem in machine learning that can drastically affect the performance of a model. Despite multi-fidelity optimization’s popularity and success, there are machine learning challenges that have not been directly addressed by existing HPOs and may require unique methodologies. Because it is so expensive to train even a small neural network on massive datasets, no work on HPO for deep neural networks on datasets, like ImageNet, has been done yet. Therefore, it is beneficial to study and analyse prevailing techniques to determine effective ways to improve them and to discover novel approaches that outperform existing ones.

In order to achieve this, we analysed the contemporary models used for HPO with the help of AutoML tools. Then we focused on Bayesian optimization and examined ways to improve a conventional Bayesian optimization framework. With the purpose of achieving this objective, evolutionary algorithms are employed to maximize the acquisition function. We compared the combination of three evolutionary algorithms with Bayesian optimization to determine which of them could outperform traditional Bayesian optimization.

The class of evolutionary algorithms is commonly used as global optimizers. They are known for their robustness in evaluating noisy objective functions and are easy to be parallelized. EAs are conceptually simple algorithms that are powerful in capturing the global optimum for complex optimization problems. They are easy to implement and do not require many constraints. Unlike gradient descent, they do not require any gradient information. EAs are not influenced by the continuity or differentiability of the objective function. Though most of the EAs require longer runtime for convergence, they tend to improve traditional optimization techniques when combined as hybrid models^63–65^.

## Methodology

The study compares HPO models that combine evolutionary algorithms with Bayesian optimization. The concept of combining BO and EA has been explored in earlier studies. STEADE is such a hybrid model, which is an evolutionary algorithm with surrogate assistance for HPO of ML models^66^. STEADE uses a mix of surrogate models (Radial Basis Function (RBF) and GP). The RBF model is used for the initial parameter space exploration, and the knowledge is then transferred to a GP-based Bayesian optimization framework that is additionally enhanced by a Differential Evolution (DE) method. Cho et al. present a hybrid method for NAS which combines evolutionary algorithms with Bayesian optimization^67^. The method, called $B^2EA$, uses two Bayesian optimization surrogate models within an EA to guide the search process: one to optimize the architecture-level hyperparameters and one to optimize the weight-level hyperparameters.

Another hybrid approach that merges BO with a genetic algorithm called GA-PARSIMONY to search for parsimony models was introduced by Martinez-de-Pison et al.^68^. Parsimony models are machine learning models that are simple and interpretable, and this method aims to find such models by combining hyperparameter optimization and feature selection. The method starts by using BO to optimize the hyperparameters of the model. Then it uses the GA-PARSIMONY algorithm to perform feature selection by searching for a subset of features that results in the best performance.

Lan et al. proposed a method that combines Bayesian optimization with evolutionary algorithms to improve the time efficiency of optimization^69^. The Bayesian optimization algorithm is used to optimize the parameters of the evolutionary algorithm, such as the population size or the mutation rate. This way, the method aims to balance the global search capability of evolutionary algorithms with the local search capability of Bayesian optimization. Our work focuses on a single surrogate model (GP) and explores the use of EAs for acquisition function optimization. The working of Bayesian optimization and details of each evolutionary algorithm used in this paper are described briefly in the following paragraphs. The pseudo-codes for each algorithm are also provided in this section.

Bayesian optimization is an optimization technique that uses a probabilistic method based on the Bayes theorem for identifying the global optimum of a black-box function. It has two major parts. The first component is a surrogate model that is probabilistic and comprises a prior distribution which represents the unknown objective function. The acquisition function is the second component, and it is optimised for selecting the next point to sample.

A surrogate model is the probability representation of the objective function, *F*. It generates a posterior probability distribution using the Bayes rule, which represents possible $F(\lambda )$ values in a candidate configuration $\lambda$. Whenever *F* is observed at a new $\lambda$, this posterior distribution is updated. The Gaussian Process (GP), which gives a mean function $\mu : \Lambda \rightarrow R$ and a definite positive covariance function $k(\lambda , \lambda '): \Lambda ^2 \rightarrow R$, also known as the kernel, is the most commonly employed surrogate model. The equation for GP is given below:

2 $$\begin{aligned} G(\lambda |\mu , \sigma ^2)= \frac{1}{\sqrt{2 \pi \sigma ^2} }e^{\frac{(\lambda -\mu )^2}{2 \sigma ^2}} \end{aligned}$$

The covariance function *k* or the Gaussian RBF kernel (also known as the squared exponential or exponentiated quadratic kernel) is defined as:

3 $$\begin{aligned} k(\lambda ,\lambda _i)=\sigma ^2 e^{-\frac{1}{2 l^2}||\lambda -\lambda _i||^2} \end{aligned}$$

where *l* is the length scale. $\lambda$ and $\lambda _i$ are *l* distance apart. The bigger the kernel function’s value, the nearer two input space points are.

We use the following equation to determine the next query point $\lambda$ :

4 $$\begin{aligned} \lambda = \mathop {\textrm{argmax}}\limits _{ \lambda \in \Lambda }\ AF(\lambda ; D) \end{aligned}$$

where *AF* is the acquisition function to maximize and *D* is the data observed so far. The popularly employed acquisition function is called Expected Improvement, EI. Expected Improvement of a single point for a Gaussian posterior is given by:

5 $$\begin{aligned} EI(\lambda ) = {\textbf {E}} [max(y-f^*, 0) | y ] \end{aligned}$$

where $f^*$ is the best function value observed so far. *y* is a random variable $\sim N(\mu (\lambda ), \sigma ^2(\lambda ))$ where *N* is normal distribution function. $\mu (\lambda )$ is the posterior mean and $\sigma (\lambda )$ is the posterior variance of *f* at $\lambda$.

6 $$\begin{aligned} EI(\lambda ) = \sigma (\lambda ) (z\phi (z) + \omega (z)) \end{aligned}$$

where $z = \frac{\mu (\lambda )-f_{max}}{\sigma (\lambda )}$. $\Phi$ and $\omega$ are the normal cdf and pdf respectively.

**Figure 1:**
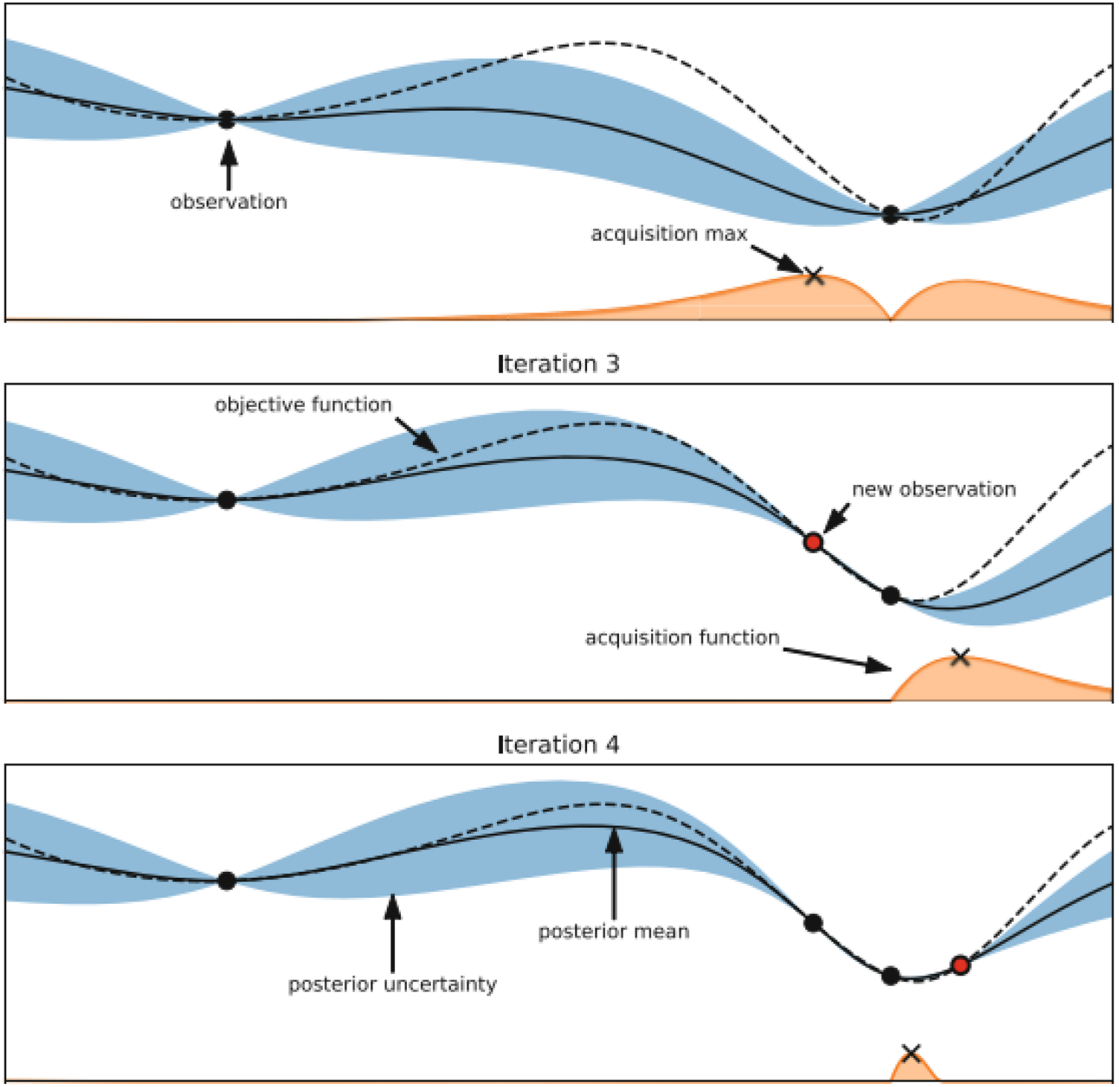
Illustration of Bayesian optimization on a 1*D* function. The graph depicts a Gaussian process approximation of the objective function and an acquisition function in the lower portion. The mean of the objective function is represented by the solid line and the dashed line represents the actual objective function. The blue region shows the predictive uncertainty. The acquisition function is represented by the orange curve. The acquisition function is maximum where the Gaussian process gives a low objective value with high uncertainty. *Note.* Adapted from Feurer and Hutter^1^.

Expected improvement comprises two parts: the first part of the sum can be improved by lowering the mean, while the other part can be improved by raising the variance. These phrases strike a compromise between exploitation and exploration. The illustration of BO is shown in Fig. 1.

In this paper, we use evolutionary algorithms like Differential Evolution, Genetic Algorithm and Evolutionary Strategy for maximizing *EI*. The foundation of every evolutionary algorithm is biological evolution, which is natural selection and continual blending of variation via recombination and mutation. New individuals (candidate solutions) are produced in each iteration (generation) by variation, typically in a stochastic way, from the existing parental individuals. Then, based on their fitness score, some individuals are chosen to become the parents of the following generation. After each generation, individuals with greater and better fitness values are generated in this manner.

A GA is a search strategy that is based on Charles Darwin’s idea of natural selection. Initialising the population, Calculating the fitness function, Selection, Crossover and Mutation are the five phases in GA. The process begins with a group of people known as the population. Each individual is denoted by a finite-length vector of components, similar to a chromosome and represents a solution in search space for a given problem. Genes are equivalent to these variable components. Thus, each chromosome (individual) is made up of multiple genes (variable components). The fitness function determines an individual’s ability to compete against others. It specifies each individual a score. This fitness score determines the likelihood of an individual being selected for the next generation.

The goal of the selection phase is to find the fittest individuals and enable them to carry on their traits to the coming generation. This is done by calculating the fitness score for each individual and selecting the highest scored pair. Physically fit individuals have a higher possibility of being selected for generating offspring. The most crucial part of the genetic algorithm is the crossover phase. A crossover site within the DNA is picked at random for each pair of parents to be mated. The genes at this point are then swapped, resulting in the creation of a totally new individual called the offspring. These new children are then included in the population. Some genes in new offspring are vulnerable to mutation, meaning that some of the bits in the DNA can be swapped. The benefits of mutation include maintaining population diversity and avoiding premature population convergence. If the population has converged, it will not generate children who are distinct from the previous generation, causing the algorithm to cease.
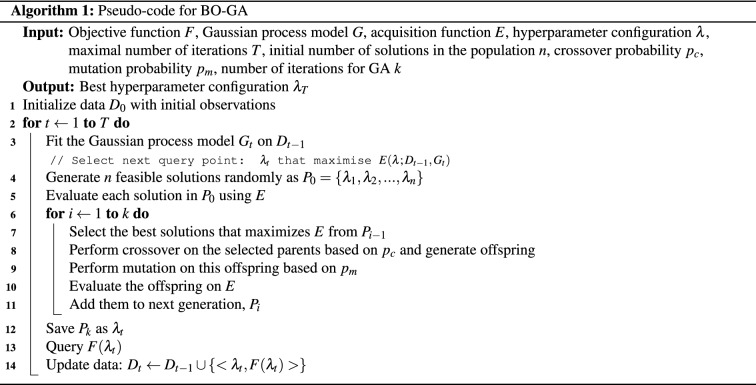


DE is an evolutionary algorithm proposed by Storn et al.^70^. DE makes minimal, if any, assumptions about the underlying optimization issue and can rapidly explore enormous design spaces. Unlike standard evolutionary algorithms, it can deal with multi-dimensional real-parameter optimization problems.

DE is also a population-based stochastic approach. Like other evolutionary algorithms, genome/chromosome is the term given to each solution. Each chromosome goes through mutation followed by recombination. Unlike the genetic algorithm that represents candidate solutions using sequences of bits, DE keeps a population of candidate solutions in the form of real-valued vectors, also called target vectors. The target vector is made of a certain number of decision variables. In each loop of the algorithm, a new donor vector is generated after mutation and a trial vector is formed out of recombination. Once the trial vectors have been generated, the best solution is selected by a greedy approach conducted among all target and trial vectors.

During mutation, DE creates new vectors called donor vectors by multiplying a third vector to the weighted difference between two population vectors. Donor vector (*V*) of a chromosome $\lambda _i$ is created as:

7 $$\begin{aligned} V = \lambda _{x_{1}} + f (\lambda _{x_{2}} - \lambda _{x_{3}}) \end{aligned}$$

where *f* is the scaling factor (0, 1 or 2), $x_{1}, x_{2}, x_{3}$ are random solutions $\in \{1,2,3,\ldots , n\}$ and $x_{1} \ne x_{2} \ne x_{3} \ne i$. Four vectors are involved in the generation of the target vector via mutation. Therefore the size of the population *n* should be greater than or equal to 4. The target vector is not involved in mutation.

Recombination is performed by binomial crossover. This step increases the diversity in the population. The trial vector is created out of recombination as follows:

8 $$\begin{aligned} u^j ={\left\{ \begin{array}{ll} v^j &{}\quad \hbox { if } r \le p_c \hbox { OR } j= \delta \\ \lambda ^j &{}\quad \hbox { if } r > p_c \hbox { AND } j \ne \delta \end{array}\right. } \end{aligned}$$

where $$p_c$$ is the crossover probability, *r* is a random number (0 or 1), $$\delta $$ is a randomly selected variable location $$\{ 1,2, . . . D\}$$ (*D* is the number of decision variables in target vector), $$ u^j$$ is the *j*th variable of trial vector, $$v^j$$ is the *j*th variable of donor vector and $$\lambda ^j$$ is the *j*th variable of target vector. $$\delta $$ assures that at least one variable from the donor vector is selected. The value of $$p_c$$ is generally set high, indicating more crossover, i.e., more variables from the donor.

During selection, a fitness function is evaluated for each offspring. The population is updated using greedy selection. If trial vector gives a better fitness score, then they are selected to the next generation. Else target vectors are added to the next generation. The selection procedure is carried out only after all solutions generate offspring.
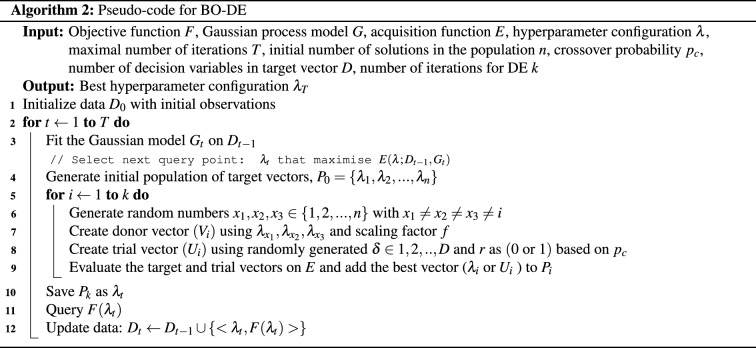


Another algorithm we use is a variant of ES. ES is a kind of evolutionary algorithm that is based on the scientific idea of natural selection in evolution. It does not use crossover like other evolutionary algorithms, instead, candidate solution modification is limited to mutation operators. The algorithm uses a population of potential solutions that are created at random. Each iteration of the method begins with an evaluation of the population of solutions, followed by truncation selection, which entails removing all but a subset of the best solutions. The remaining solutions (parents) are each utilised to generate a set of new candidate solutions (mutation) that replace or compete with the parents for a place.

This approach has several variations. The number of parents chosen per iteration is measured in *p* and the number of offspring is *o*. One of the variations is represented as (*p*, *o*)-ES, a version where children take the place of parents. Another variation is represented as (*p* + *o*)-ES where children and parents are added to the population.

CMA-ES is the most powerful variation of ES^71^. For an initial population $P=\{\lambda _1, \lambda _2,\ldots \lambda _n\}$ of size *n*, the major differences between CMA-ES and classic ES are:

1. Offsprings are not generated by mutation of a single individual but by the weighted mean of the current population:
  9 $$\begin{aligned} \lambda _{j} = \mu + \sigma {} y_{j} \end{aligned}$$
  where $\sigma$ $( > 0)$ is step-size, *y* is a random vector for $j= 1, 2,\ldots , n$ and
  10 $$\begin{aligned} \mu = \frac{1}{\sum _{j=1}^{m}w_j} \sum _{j=1}^{m} w_j \lambda _j, \ \ \ \ w_j >0 \end{aligned}$$
  $\mu$ gives the weighted mean, $w_j$ is the set of positive weight coefficients for recombination and $\lambda _j$ is the set of *m* best individuals out of the current population.
2. Unlike standard ES, *y* is not chosen to be independent of *j*, but chosen such that:
  11 $$\begin{aligned} y \sim N(0,C) \end{aligned}$$
3. *C* is updated at each iteration $(t+1)$ using a rank-1 update:
  12 $$\begin{aligned} C^{(t+1)}=(1-\alpha )C^{(t)} + \alpha Q Q^T \end{aligned}$$
  where *Q* is the cumulative path parameter, initialised as 0 and updated using the equation:
  13 $$\begin{aligned} Q^{(t+1)}=(1-\beta )Q^{(t)} + \beta \sqrt{\beta (2-\beta )m} \Big ({\frac{\mu ^{(t+1)} - \mu ^{(t)}}{\sigma ^{(t)}}} \Big ) \end{aligned}$$
  $\alpha , \beta$ are learning rates $\in (0,1]$.
4. Step size control is integrated into CMA-ES. That is, $\sigma$ in Eq. (9) is chosen in a way that prevents the population from converging prematurely.

The major advantage of CMA-ES over other evolutionary algorithms is that the population size can be freely chosen. There is no inherent need to use large population sizes.
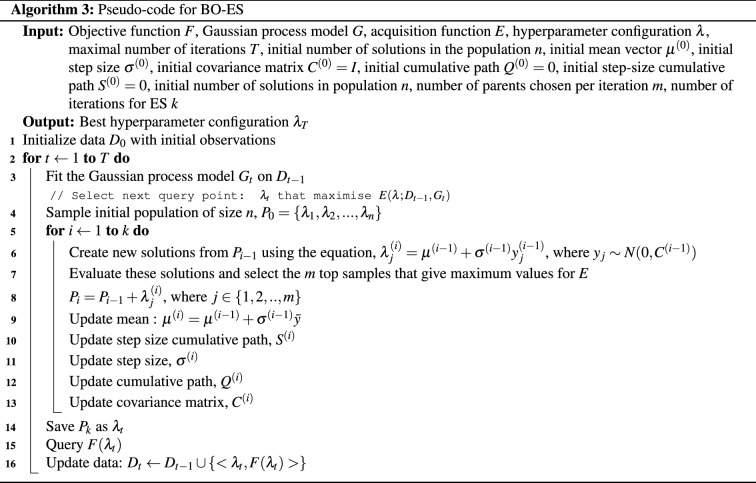


The time complexity of BO algorithm with a Gaussian process surrogate model is $O(n^3)$, where *n* is the number of hyperparameter values^72^. GA has an asymptotic run time of $O(n^2)$. For DE and CMA-ES, it is $O(n^3)$^73^. The computational complexity of BO-GA, BO-DE and BO-ES are $O(n^3)$.

## Results and discussion

As part of the study, we have conducted three sets of experiments, all of which uses the same sets of data and are run on the same configuration. We use an Intel(R) Xeon(R) Gold 6240R CPU @ 2.40GHz with 1 NVIDIA Tesla V100 GPU. The datasets used include CIFAR-10, MNIST, SVHN and CALTECH-101.

CIFAR-10 comprises 6000 images belonging to 10 different classes. It includes images of aeroplanes, birds, frogs, cats, deer, horses, cars, ships, dogs and trucks. MNIST consists of 60,000 grayscale square images ($28 \times 28$ pixels) of handwritten single digits from 0 to 9. Street View House Number dataset contains 6,00,000 RGB images ($32\times 32$ pixels) of printed digits from 0 to 9 clipped from photographs of house number plates. CALTECH dataset has 101 object categories, with about 40 to 800 images belonging to each category ($300 \times 200$ pixels).

### Setup 1

Experiment setup 1 aims to analyse four HPO methods—Random Search (RS), BO, Hyperband and GA with the help of AutoML systems. AutoML systems with different optimizers are evaluated based on their performance in image classification problems. AutoKeras and TPOT are used in this experiment.

AutoKeras is an open-source software library for AutoML. It is built on top of the Keras deep learning library and can be used to automatically search for the best model for a given dataset. AutoKeras includes a preprocessing step that automatically detects the type of problem and the format of the input data and then selects the appropriate ML models to try. It also uses a technique called NAS to search for the best neural network architecture for a given dataset. It starts by generating a large number of randomly initialized neural network architectures and then trains and evaluates each one on the dataset. The best-performing architectures are then selected and used to generate new architectures through mutations and crossovers. This process is repeated until a satisfactory architecture is found or a pre-defined stopping criterion is met. AutoKeras also supports transfer learning, which allows the user to fine-tune a pre-trained model on a new dataset. This can significantly speed up the training process and improve performance on small datasets. AutoKeras includes a feature for HPO. By default, AutoKeras uses random search, but it also supports other HPO methods like Bayesian optimization and Hyperband. Users can specify which method to use. Finally, it outputs the best architecture or ML model with the best set of hyperparameters for a given dataset.

TPOT (Tree-based Pipeline Optimization Tool) is another open-source software library for AutoML, built on top of the scikit-learn library. It can be used to automatically search for the best pipeline of preprocessing steps and ML models for a given dataset. TPOT uses genetic programming to search for the best pipeline. It starts by generating a population of randomly initialized pipelines and then trains and evaluates each one on the dataset. The best-performing pipelines are then selected and used to generate new pipelines through mutations and crossovers. This process is repeated until a satisfactory pipeline is found or a pre-defined stopping criterion is met. TPOT includes a wide range of preprocessing steps, such as feature scaling and feature selection, as well as ML models, such as linear regression, decision trees, and neural networks. The HPO in TPOT is performed using GA. Last, it returns the best ML model for the specified dataset with the best combination of hyperparameters.

**Figure 2:**
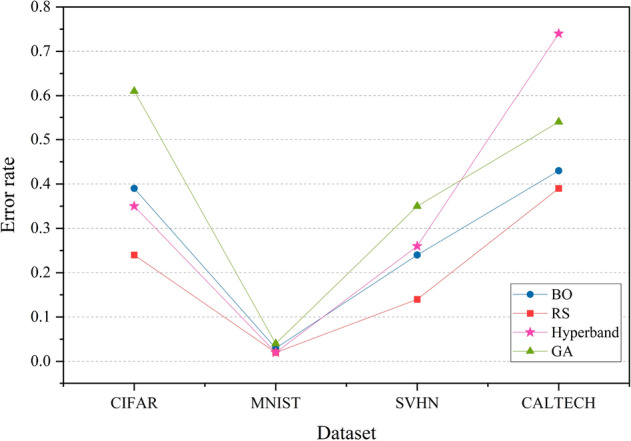
Test set classification error plot of AutoKeras and TPOT. The figure shows the average test error rate across the datasets—CIFAR, MNIST, SVHN and CALTECH. The HPO techniques compared in the graph are BO, RS, Hyperband and GA. AutoKeras is run 3 times—using BO, RS and Hyperband for HPO for each dataset. TPOT is run once for each dataset and it uses GA for HPO.

**Figure 3:**
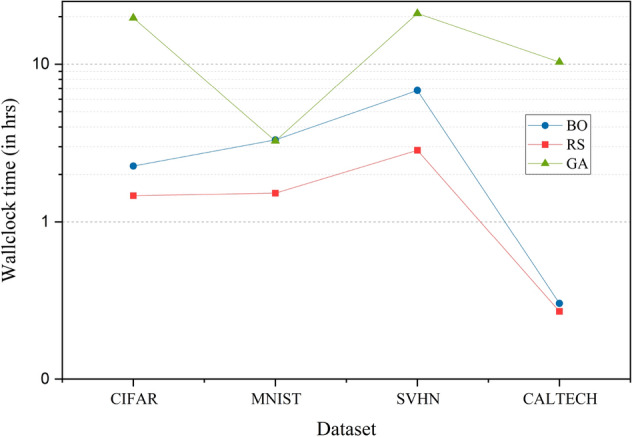
Wallclock time comparison plot of AutoKeras and TPOT across the datasets—CIFAR, MNIST, SVHN and CALTECH. The HPO techniques compared in the graph are BO, RS, Hyperband and GA. AutoKeras is run 3 times—using BO, RS and Hyperband for HPO for each dataset. TPOT is run once for each dataset and it uses GA for HPO.

The comparison of test error rates and wallclock time for experiment setup 1 are shown in Figs. 2 and 3. Figure 2 represents a line graph showing the error rates obtained for the classification of CIFAR, MNIST, SVHN and CALTECH data using BO, RS, Hyperband and GA for hyperparameter optimization. Overall, random search gives better results than the other three HPO techniques in terms of test error rate for all four datasets.

The green line in the graph represents genetic algorithm. It is observed that genetic algorithm has the highest error rates implying low classification accuracy for three of the datasets. The blue line depicting the performance of Bayesian optimization shows that it produces a better outcome compared to GA in all four cases. Hyperband performs better than GA in most cases, but for CALTECH data, it gives an error rate of 0.7, indicating a very low accuracy. Random search, presented by the red line, has the lowest error rates for all classification problems.

Bayesian optimization is not the best optimizer for the image classification datasets we chose. So we looked for ways to enhance BO. We tried to use other optimization algorithms for acquisition function maximization. We concentrated on Evolutionary Algorithms for this purpose. Since we already observed the performance of GA, we will compare the performance of DE with BO in setup 2. And finally, in setup 3, we will examine if the combinations could enhance the efficiency of conventional BO.

Figure 3 shows datasets used on the x-axis and the time taken by AutoKeras (with BO, RS and Hyperband used for HPO) and TPOT (uses GA for HPO) on the y-axis. The graph is a semi-logarithmic plot that uses a logarithmic scale on the y-axis to indicate the variation in wallclock time from seconds to hours.

It is observed from the graph that TPOT requires hours-to-days to complete the image classification tasks. Random search takes rather less time compared to BO for all datasets. Hyperband is not included in the comparison because it uses early stopping criteria and requires very less time in contrast to other optimization techniques. TPOT is recommended only in the case where time is not considered a constraint.

### Setup 2

To focus on BO and look for possibilities to improve its performance, we carried out the second experiment that compared optimizer packages that use BO and EA. Since the first experiment included GA, we used a package that employed a different evolutionary algorithm. So we selected DEHB that used differential evolution, and analysed its performance in contrast to BO. The datasets used for this analysis are also the same. The packages are used to optimize the hyperparameters of an AlexNet, a deep learning architecture with 8 layers. It consists of 5 convolution layers and a mix of max-pooling layers. There are three layers that are fully connected. AlexNet introduced the use of non-saturating ReLU function as activation function, which resulted in improved training accuracy over tanh function and sigmoid. Two dropout layers are used. The output layer uses a Softmax activation function.

AlexNet is a fast GPU execution of a convolutional neural network. AlexNet won the ImageNet Large Scale Visual Recognition Challenge in 2012. The main conclusion of the original research on AlexNet was that the model’s depth was critical for its high performance, which was computationally costly but made possible by the use of GPUs during training^74^.

**Figure 4:**
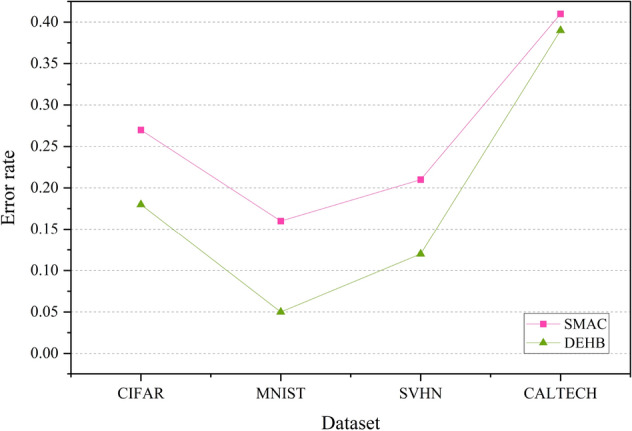
Test set classification error plot of an AlexNet model that uses the HPO packages—SMAC and DEHB. The figure represents the average test error rate across the datasets—CIFAR, MNIST, SVHN and CALTECH (based on 20 iterations). SMAC is a BO package and DEHB combines DE and Hyperband. The results are obtained using the Wilcoxon Rank-Sum test on samples from 20 iterations with a statistical significance of p $<0.05$.

The experiment considers an AlexNet model and 4 of its hyperparameters. Table 1 gives the list of hyperparameters and the range of values used for tuning. The optimizers used in the experiment are Sequential Model-based Algorithm Configuration (SMAC) and Differential Evolution Hyperband (DEHB). The main core of SMAC combines Bayesian optimization with a mechanism to quickly determine which of the two configurations is the better performer. It uses a Random Forest (RF) surrogate model. On the other hand, DEHB combines the advantages of the popular bandit-based HPO method Hyperband and the evolutionary search approach of Differential Evolution. Figure 4 illustrates the comparison of the two HPO packages based on error rates obtained using the Wilcoxon Rank-Sum test on samples from 20 iterations with a statistical significance of p-value $< 0.05$. From Fig. 4, it is clearly noticeable that the green line representing DEHB has lower error rates for all four datasets compared to the pink depicting SMAC. DEHB with the advantages of both Hyperband and Differential Evolution proves to be more promising than SMAC. This outcome gave us the notion that combining DE and BO might improve the performance of BO.

**Figure 5:**
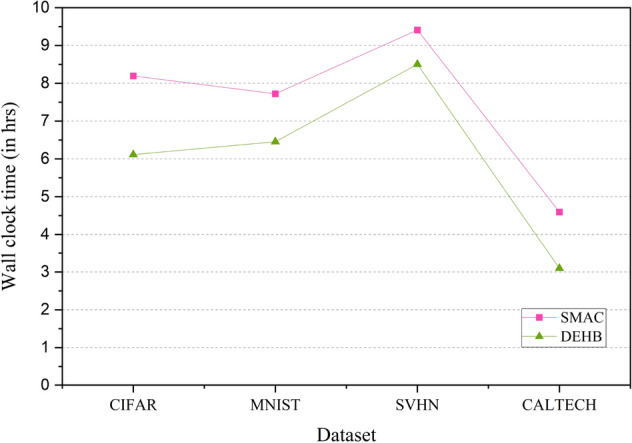
Wallclock time comparison plot of AlexNet model across the datasets—CIFAR, MNIST, SVHN and CALTECH. The HPO packages used here are SMAC and DEHB. SMAC is a BO package and DEHB combines DE and Hyperband.

Figure 5 shows the wall clock time comparison of the AlexNet model on CIFAR, MNIST, SVHN and CALTECH. The model uses SMAC and DEHB for HPO. The x-axis represents the datasets and y-axis represents the time taken by SMAC and DEHB. It is observed from the graph that DEHB takes lesser time compared to SMAC for all four classification problems.

**Table 1:** Hyperparameter values for tuning.

Sl. no.	Hyperparameter	Range
1	Learning rate	[0.0001–1.0]
2	Batch size	[32–1024]
3	Epochs	[10–100]
4	Momentum	[0.9–0.999]

### Setup 3

Setup 3 attempts to compare various combinations of Bayesian optimization with evolutionary algorithm models with traditional BO, random search and genetic algorithm. The HPO models we compare here are RS, GA, BO, BO-DE, BO-CMAES and BO-GA in terms of error rate and time efficiency. The test considers an AlexNet model and four of its hyperparameters for optimization.

**Figure 6:**
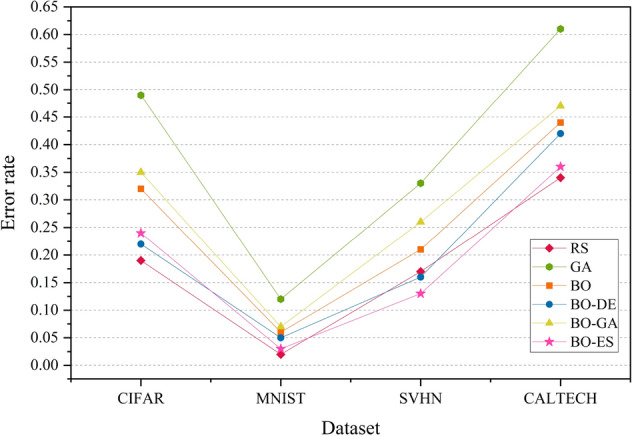
Test set classification error plot of an AlexNet model that uses RS, GA, a BO framework for HPO with L-BFGS, BO-DE, BO-GA and BO-ES. The figure depicts the average test error rate across the datasets—CIFAR, MNIST, SVHN and CALTECH (based on 20 iterations). The results are obtained using the Friedman test with a statistical significance of p $<0.05$.

The experiment is executed by implementing the BO variants on the same set of data. We compare the three HPO models that employ DE, GA and CMA-ES for acquisition function maximization against traditional BO, RS and GA. The results are analysed in terms of the error rate obtained using the Friedman test on samples from 20 iterations with a statistical significance of p-value $< 0.05$ and is shown in Fig. 6.

All variants of BO used here are compared to the standard Bayesian optimization. Conventional BO usually uses random optimizers for maximizing EI. Here we use a combination of random sampling (cheap) and the ‘L-BFGS-B’ (Limited-memory Broyden–Fletcher–Goldfarb–Shanno algorithm with bound constraints) optimization method. L-BFGS-B is a variant of L-BFGS that uses a limited amount of computer memory to approximate the L-BFGS algorithm. It is a second-order quasi-Netwon method and has a faster convergence rate.

Figure 6 shows that the overall efficiency of BO combined with CMA-ES has better classification accuracy compared to combinations of BO with other evolutionary algorithms in most cases. BO combined with DE produces better results than GA. The graph also proves that the performance of standard BO can be improved by using DE and CMA-ES for acquisition function maximization. RS gives the best accuracy for most datasets. This is because RS performs well in small hyperparameter spaces. Since we have considered only 4 hyperparameters (low-dimension search space), RS delivers good performance. But this cannot be guaranteed for higher dimensional hyperparameter search spaces.

**Figure 7:**
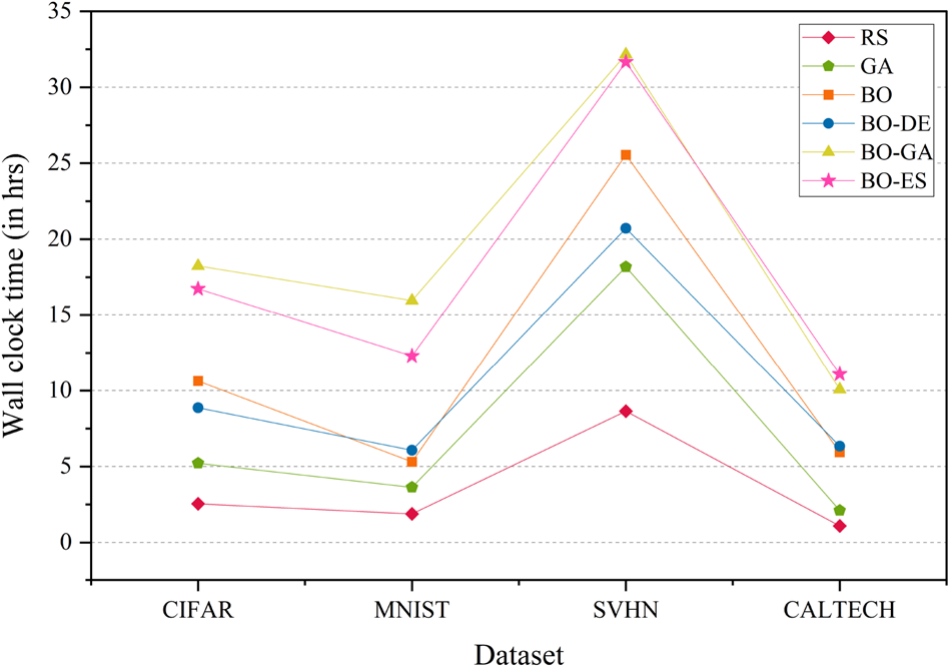
Wallclock time comparison plot of AlexNet model across the datasets—CIFAR, MNIST, SVHN and CALTECH. The model uses RS, GA, a BO framework for HPO with L-BFGS, BO-DE, BO-GA and BO-ES.

Figure 7 shows the wall clock time comparison of the AlexNet model on CIFAR, MNIST, SVHN and CALTECH. The model uses a BO framework for HPO with L-BFGS, DE, GA and ES for acquisition function optimization. BO-DE and BO perform better than BO-ES and BO-GA in terms of run-time. RS and GA took less time compared to other HPO models. RS may not be time-efficient in high-dimensional hyperparameter search space. In a high-dimensional space, the chance of finding the optimal hyperparameters by chance becomes very small, and the search process can become extremely inefficient and time-consuming. In such cases, RS may require a large number of trials to cover the entire search space, which can be computationally expensive and time-consuming. This is because RS generates random combinations of hyperparameters without considering any prior information or structure in the search space, leading to a lot of redundant trials and a low probability of finding the optimal hyperparameters. GA performs poorly in all cases.

**Figure 8:**
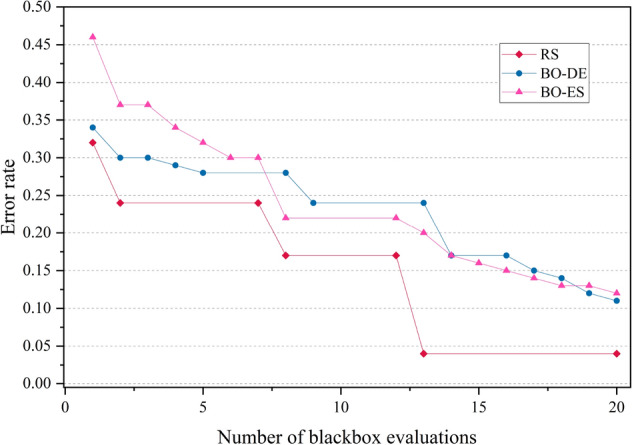
Comparison of how error rates of AlexNet evolve with the number of function evaluations for RS, BO-ES and BO-ES on CIFAR data.

**Figure 9:**
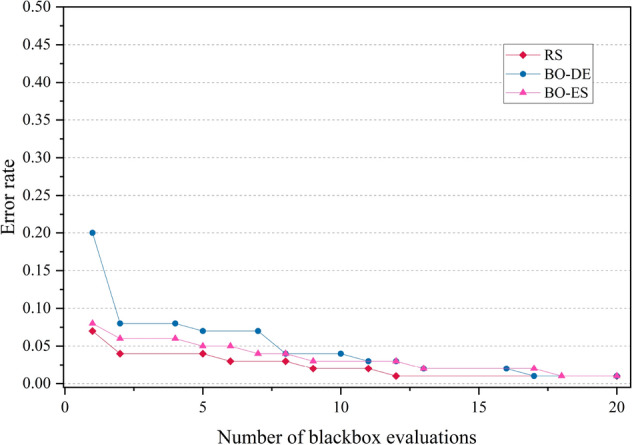
Comparison of the error rates of AlexNet against the number of function evaluations for BO-ES, BO-ES, and RS using MNIST data.

**Figure 10:**
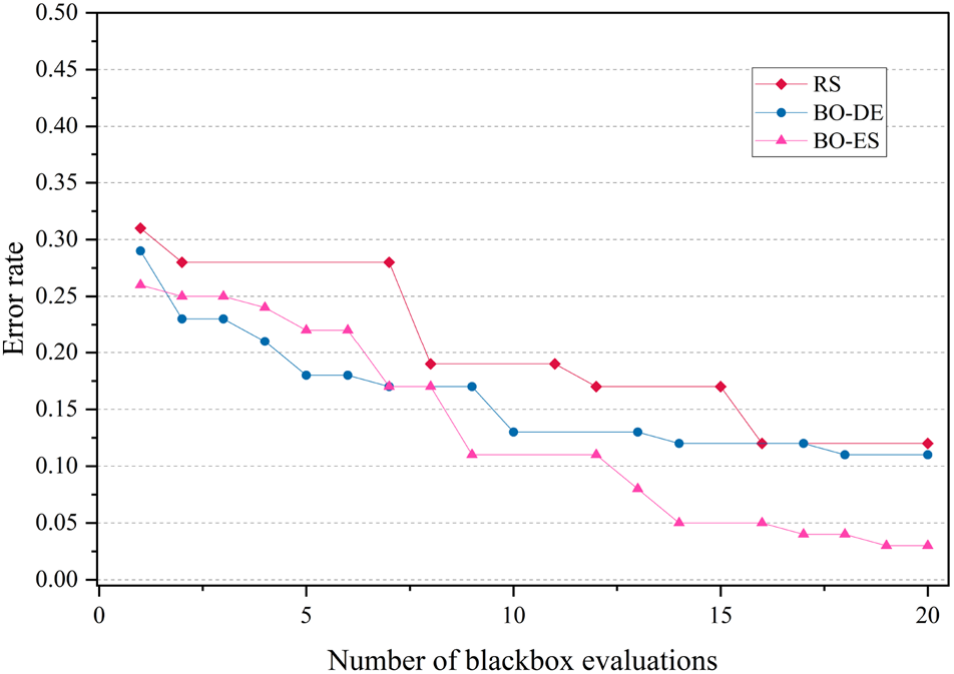
Comparison of the error rates of AlexNet against the number of function evaluations for BO-ES, BO-ES, and RS using SVHN data.

**Figure 11:**
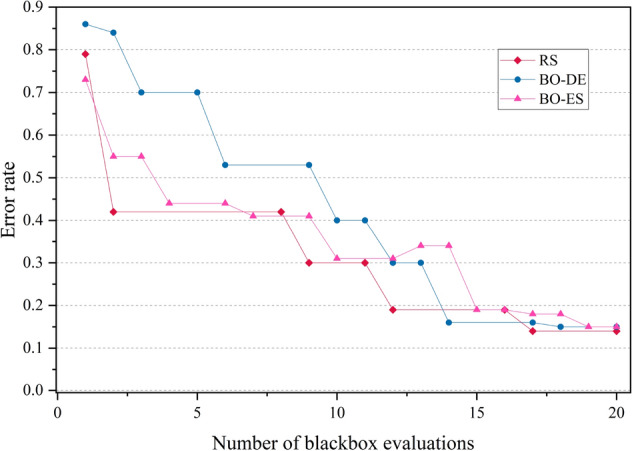
Comparison of how error rates of AlexNet evolve with the number of function evaluations for RS, BO-ES and BO-ES on CALTECH data.

The error rate versus number of blackbox evaluation graphs are given in Figs. 8, 9, 10 and 11 corresponding to CIFAR, MNIST, SVHN and CALTECH respectively. We have compared the best performing three models: RS, BO-DE and BO-ES. Only successful error rate reductions are depicted in the graph. The downward trend in the graphs mean that over time, more promising regions are being examined more frequently. A flat trend indicates very little learning from prior experiences. BO-DE and BO-ES finds more number of better configuration than RS in all figures. We have considered average of the test error rates from 20 iterations when plotting Fig. 6.

### Setup 4

Similar to setup 3, setup 4 compares RS, GA, BO, BO-DE, BO-CMAES and BO-GA. But the experiment is carried out on a Densenet model considering four of its hyperparameters. The list and range of the hyperparameters are in Table 1.

DenseNet-121 is a convolutional neural network (CNN) architecture for image classification. It consists of the following components: (1) Initial Convolution: The network starts with a standard convolutional layer to extract low-level features. (2) Dense Blocks: The core of DenseNet121 is made up of dense blocks, which contain multiple layers with different numbers of filters and each layer is connected to all preceding layers. This allows the network to learn more robust and diverse features, and improve feature reuse. (3) Transition Layers: Between each dense block, a transition layer is used to reduce the number of filters, and also to perform dimensionality reduction by using pooling to reduce the spatial dimensions. (4) Final Classification: The network ends with a standard fully connected layer and a softmax activation function to produce the final class probabilities. In total, DenseNet-121 has 120 convolution layers, 4 average pooling layers and 1 fully connected layer. In this architecture, features from previous layers are concatenated with the features of the current layer, leading to increased feature reuse and reduced risk of overfitting. The architecture is well suited for tasks involving large amounts of image data and has achieved state-of-the-art results on benchmarks such as ImageNet^75^.

**Figure 12:**
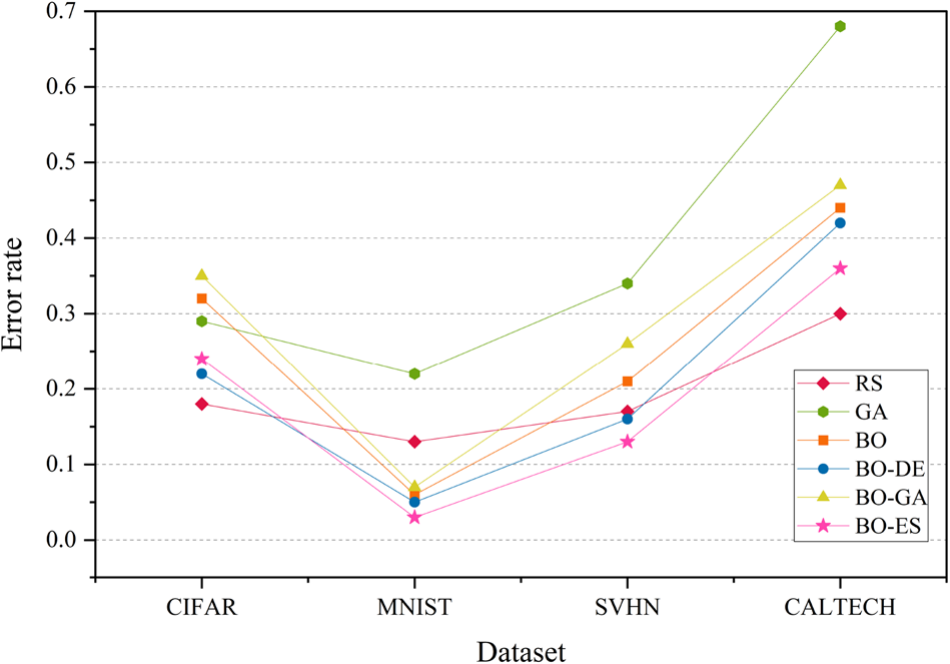
Test set classification error plot of DenseNet model that uses RS, GA, a BO framework for HPO with L-BFGS, BO-DE, BO-GA and BO-ES. The figure depicts the average test error rate across the datasets—CIFAR, MNIST, SVHN and CALTECH (based on 20 iterations). The results are obtained using the Friedman test with a statistical significance of p $<0.01$.

Figure 12 illustrates the results in terms of the error rate determined by the Friedman test on samples from 20 iterations with a statistical significance of p-value $< 0.01$. RS gives the best accuracies for CIFAR and CALTECH data while BO-ES gives better results for MNIST and SVHN datasets. As for AlexNet, BO-ES and BO-DE perform better than standard BO. GA and BO-GA deliver the lowest accuracies for most datasets.

**Figure 13:**
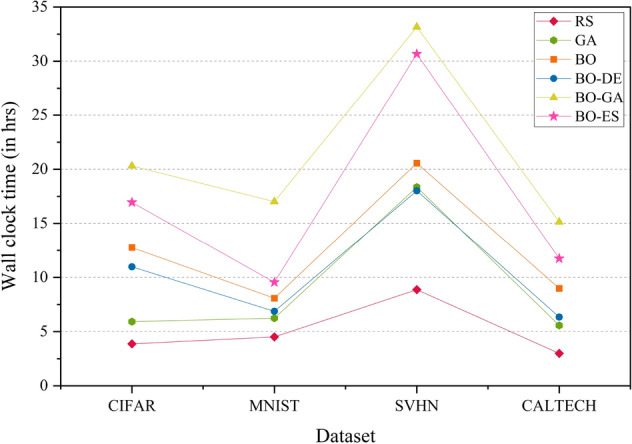
Wallclock time comparison plot of DenseNet model across the datasets—CIFAR, MNIST, SVHN and CALTECH. The model uses RS, GA, a BO framework for HPO with L-BFGS, BO-DE, BO-GA and BO-ES.

The wall clock time comparison of the DenseNet model on CIFAR, MNIST, SVHN, and CALTECH is presented in Fig. 13. RS takes the least time for all datasets. As mentioned earlier, the time efficiency of RS depends on the dimensionality of hyperparameter space. BO-GA and BO-ES take more time than standard BO and BO-DE.

**Figure 14:**
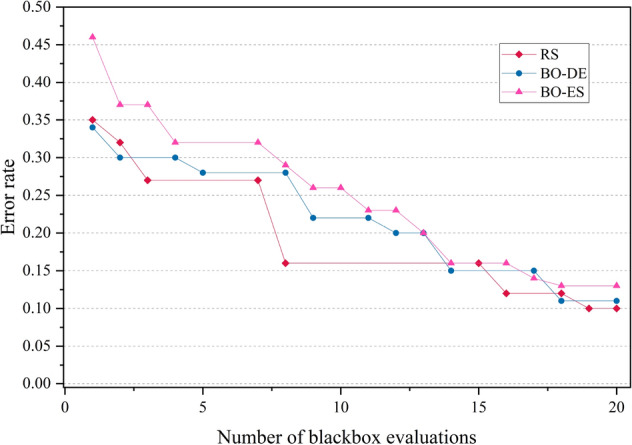
Comparison of how error rates of DenseNet evolve with the number of function evaluations for RS, BO-ES and BO-ES on CIFAR data.

**Figure 15:**
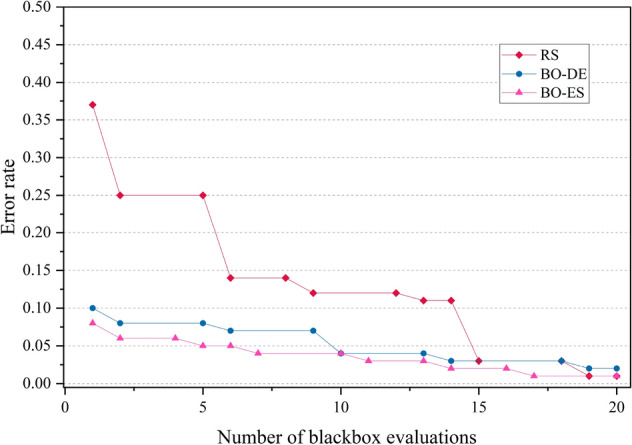
Comparison of the error rates of DenseNet against the number of function evaluations for BO-ES, BO-ES, and RS using MNIST data.

**Figure 16:**
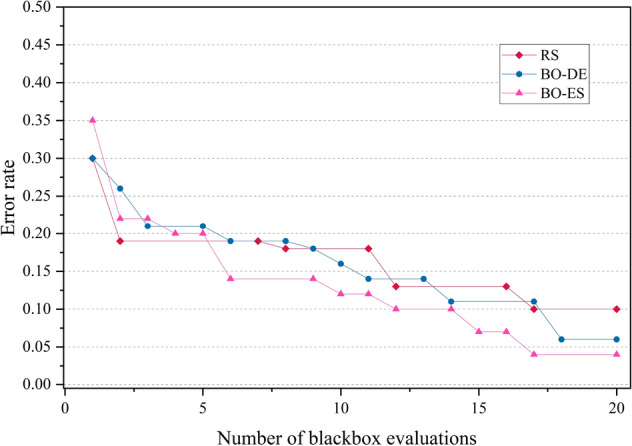
Comparison of the error rates of DenseNet against the number of function evaluations for BO-ES, BO-ES, and RS using SVHN data.

**Figure 17:**
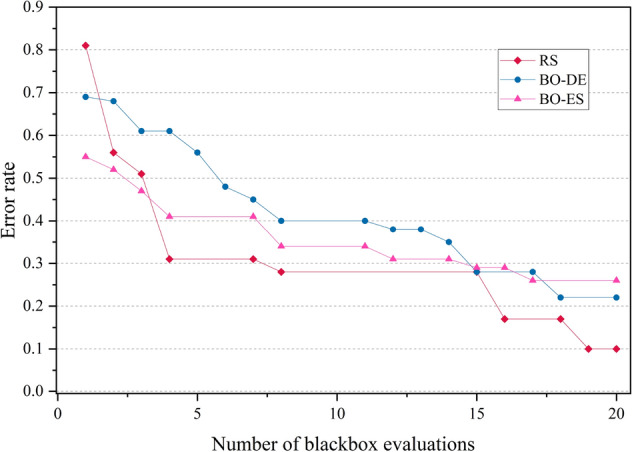
Comparison of how error rates of DenseNet evolve with the number of function evaluations for RS, BO-ES and BO-ES on CALTECH data.

Figures 14, 15, 16 and 17 depicts the development of optimization techniques on CIFAR, MNIST, SVHN and CALTECH datasets. The three models RS, BO-DE, and BO-ES that perform the best have been compared. The graph only shows successful error rate reductions. The steady decline in graphs indicate that more promising configurations are being looked at more often over time. In all figures, BO-DE and BO-ES discover more instances of better configuration than RS. When plotting Fig. 12, we took into account the mean test error rate of 20 iterations.

## Conclusion

Hyperparameters of ML models must be tuned to fit particular datasets before being deployed to practical problems. However, the volume of created data has substantially grown in practice. And manually setting all hyperparameters is tremendously resource-intensive. Therefore, it has become critical to optimise hyperparameters automatically. We have seen the efficacy of AutoML systems in handling large-scale image classification datasets. We have also observed the performance of SMAC and DEHB packages for hyperparameter optimization. In particular, we compared different HPO models on AlexNet and DenseNet architectures. Out of the HPO frameworks compared in this study, it is examined that the efficiency of BO-CMAES and BO-DE proves it to be worth adopting in AutoML systems. The results from the paper can be extended to other models and datasets. The number of hyperparameters can also be scaled along with the range of values considered for tuning. The above experiments are carried out for 20 iterations. For better results, the number of iterations can be increased. As future work, we plan to apply this comparison of algorithms to a different application. We also plan to conduct similar experiments for real-time image classification problems.

## Data Availability

Data underlying the results presented in this paper are publicly available and can be downloaded from Kaggle.
